# Carbon Nanohorns Promote Maturation of Neonatal Rat Ventricular Myocytes and Inhibit Proliferation of Cardiac Fibroblasts: a Promising Scaffold for Cardiac Tissue Engineering

**DOI:** 10.1186/s11671-016-1464-z

**Published:** 2016-06-04

**Authors:** Yujing Wu, Xiaoli Shi, Yi Li, Lei Tian, Rui Bai, Yujie Wei, Dong Han, Huiliang Liu, Jianxun Xu

**Affiliations:** Department of Cardiology, General Hospital of Chinese People’s Armed Police Forces, Yongding Road, Haidian District, Beijing, 100039 China; National Center for Nanoscience and Technology, Beijing, 100080 China

**Keywords:** Carbon nanohorns, Cardiac tissue engineering, Neonatal rat ventricular myocytes, Cardiac fibroblasts, Maturation

## Abstract

Cardiac tissue engineering (CTE) has developed rapidly, but a great challenge remains in finding practical scaffold materials for the construction of engineered cardiac tissues. Carbon nanohorns (CNHs) may be a potential candidate due to their special structure and properties. The purpose of this study was to assess the effect of CNHs on the biological behavior of neonatal rat ventricular myocytes (NRVMs) for CTE applications. CNHs were incorporated into collagen to form growth substrates for NRVMs. Transmission electron microscopy (TEM) observations demonstrated that CNHs exhibited a good affinity to collagen. Moreover, it was found that CNH-embedded substrates enhanced adhesion and proliferation of NRVMs. Immunohistochemical staining, western blot analysis, and intracellular calcium transient measurements indicated that the addition of CNHs significantly increased the expression and maturation of electrical and mechanical proteins (connexin-43 and N-cadherin). Bromodeoxyuridine staining and a Cell Counting Kit-8 assay showed that CNHs have the ability to inhibit the proliferation of cardiac fibroblasts. These findings suggest that CNHs can have a valuable effect on the construction of engineered cardiac tissues and may be a promising scaffold for CTE.

**Electronic supplementary material:**

The online version of this article (doi:10.1186/s11671-016-1464-z) contains supplementary material, which is available to authorized users.

## Background

Acute myocardial infarction can lead to sudden anoxia and ischemic injury in cardiomyocytes, impairing ventricular function and ultimately leading to heart failure at the end stage. Cardiac tissue engineering (CTE) compared with traditional heart transplantation will be a promising therapeutic strategy for end-stage heart failure in the future, and this approach is developing rapidly. Scaffolds play a very important role in CTE. The optimal scaffold can improve the extracellular microenvironment, provide extracellular signals, promote cell survival, and regulate cell differentiation and maturation [1].

Collagen and other natural biomaterials in the extracellular matrix have roles as structural proteins and have a close affinity to cells, but they do not do well in cell retention [2, 3]. Artificial biomaterials, particularly nanomaterials due to their special structures and electrical properties, have attracted significant research interest in the construction of engineered cardiac tissue.

Carbon nanohorn (CNH) is a horn-shaped graphitic tubule with a diameter of 2 to 5 nm. Thousands of CNHs usually form a spherical structure with a diameter of 50 to 100 nm. In contrast to other carbon nanomaterials, no metal catalyst is used in the CNH synthesis processes. Metal catalysts are the major impurity inducing biological toxicity through an oxidative stress response. CNHs have been shown to possess low toxicities in vivo and in vitro through various exposure pathways for toxicological assessments [4]. In previous studies, several electric nanomaterials, including carbon nanotubes [5, 6], graphene [7–9], and gold nanoparticles [10, 11], have been reported to promote cardiomyocyte maturation or the differentiation of stem cells into cardiac myocytes.

The hyperplasia and fibrosis of cardiac fibroblasts are closely related to cardiac remolding, a pathological consequence of post-myocardial infarction [12, 13]. It has been reported that carbon nanomaterials govern selective cell-specific behaviors. For example, carbon nanotubes increase cardiomyocyte proliferation and maturation but do not change the cardiac fibroblast viability or proliferation [14, 15]. Thus, there is a need to explore the effect of carbon nanohorn-collagen (CNH-Col) biomaterials on cardiomyocytes and cardiac fibroblasts.

The purpose of our study was to assess the effect of CNH-Col material on neonatal rat ventricular myocytes (NRVMs) for CTE applications. In this study, we utilized CNH-Col material as a NRVM growth substrate for comparison with collagen substrates. We found that CNH-Col substrates not only can promote differentiation and maturation of NRVMs but also can inhibit the viability and proliferation of cardiac fibroblast and may further reduce cardiac remolding.

## Methods

### Preparation of CNH-Col Composite Substrates

CNHs were obtained from NEC Corporation; they were produced by high-power CO_2_ laser ablation at room temperature in Ar. The carbon source was a pure graphite target without any metallic catalyst. The collagen was extracted from rat tails and diluted with acetic acid aqueous solution (pH = 3) to 3 mg/ml. CNHs were dispersed with the collagen in an ultrasonic ice bath for 5 min/ml. After being centrifuged (12,000×*g*, 10 min), the CNH-Col mixtures were used as stock solutions at a concentration of 1 mg/ml. The final target solutions were at concentrations of 0, 0.05, 0.1, 0.15, and 0.2 mg/ml. Then, the CNH-Col solutions were deposited onto glass coverslips (150 μl/cm^2^) and dried in a vacuum oven at 60 °C overnight. Finally, the CHN-Col substrates were irradiated under ultraviolet light for sterilization for 3 h.

### Characterization of CNH-Col Material

#### Morphological Observations

The CNH-Col solution was dropped onto a Cu grid coated with a thin carbon film for transmission electron microscopy (TEM, Hitachi H-7500) observations using a Tecnai F20 instrument (200 kV). The surface morphology of the CNH-Col and collagen was examined with an atomic force microscope (AFM) (MultiModeVII, Bruker, USA) using ScanAsyst in air mode.

#### Electroactivity and Conductivity Measurement

The electroactivity and conductivity of CNH-Col and collagen were estimated using an electrochemical workstation. Briefly, the CNH-Col solution, having a CNH concentration of 0.05 and 0.1 mg/ml, and collagen solution were added to a container (length 8 mm, diameter 5 mm), respectively, and inserted between two copper current collectors to form a sandwich-like structure. Then, the assembled structure was connected to the electrochemical workstation instrument. Current-voltage characteristics of each sample were recorded by sweeping the applied voltage from −0.1 to 0.1 V. The electrical conductivity (*σ*) was calculated by the following equation (*ρ* electrical resistivity, *V* voltage, *I* current, *S* cross-sectional area, and *L* length of measured material): *σ* = 1/*ρ* = *I*/*V* × *L*/*S*.

Cyclic voltammetry readings were recorded between the potentials of −0.8 and 0.8 V at a sweep rate of 100 mV/s for multiple cycles by a multichannel potentiostat (VMP3, Biologic, Knoxville, TN) using potentials. All of the data were recorded under a stable state.

### Cell Isolation and Cultivation

According to a protocol accepted by the Institutional Animal Care and Use Committee of the Chinese Academy of Military Medical Science (Beijing, China), NRVMs and cardiac fibroblasts were isolated from 1-day-old Sprague Dawley rats as previously described [16–19]. NRVMs and cardiac fibroblasts were seeded onto substrates (10^6^/cm^2^ and 6 × 10^3^/cm^2^ cells, respectively) and cultured in DMEM (Gibco, USA) containing 15 % FBS (Invitrogen, Carlsbad, CA, USA) at 37 °C, 5 % CO_2_. The culture medium was refreshed every other day.

### Cell Viability and Proliferation Measurement

At days 1 and 3, the LIVE/DEAD Viability/Cytotoxicity Kit (Invitrogen, Grand Island, NY) and Cell Counting Kit-8 (CCK-8) assay (Beyotime, Shanghai, China) were utilized following the manufacturers’ instructions. NRVMs were stained with calcein-AM/ethidium homodimer to detect live/dead cells, respectively. CCK-8 reagent was added to a culture medium of NRVMs and cardiac fibroblasts. After 3 h of incubation, the absorbance values at 450 nm were measured using an enzyme-linked immunosorbent assay microplate reader (Molecular Devices, Sunnyvale, CA).

### Cell Adhesion Measurement

The attached cells were quantified to evaluate cell adhesion on CNH-Col and collagen substrates. Briefly, NRVMs were seeded on CNH-Col and collagen substrates (6 × 10^5^/cm^2^ cells). After 12 and 24 h of incubation at 37 °C, 5 % CO_2_, detached cells were carefully washed away by rinsing with PBS three times, and the attached cells were counted in 10 randomly selected fields for each group under a phase-contrast microscope.

### Immunofluorescence Staining and Confocal Microscopy

After 3 and 7 days of cultivation, the NRVMs were fixed in 4 % formaldehyde for 30 min. Then, the cells were permeabilized in 0.3 % Triton-100 for 30 min and blocked with 2 % goat serum for 30 min. The following primary antibodies were used to stain cells at 4 °C overnight: mouse monoclonal anti-troponin T (TnT, 1:100, Abcam), mouse monoclonal anti-alpha-actinin (α-actinin, 1:100, Abcam), rabbit polyclonal anti-connexin-43 (Cx-43, 1:1000, Abcam), and rabbit anti-N-cadherin (NC, 1:200, Abcam). The relative immunofluorescence Alexa Fluor 488- and Alexa Fluor 548-conjugated secondary antibodies (1:500, Invitrogen) were conjugated with the primary antibody for 2 h of cultivation at 37 °C. Finally, the cells were counterstained with Hoechst33258 4-6-diamidino-2-phenylindole (DAPI) in D-PBS (Sigma, 1:1000) and analyzed under a Zeiss confocal microscope with Volocity Demo 6.1.1.

### Western Blot Analysis

After 3 and 7 days of NRVM cultivation on CNH-Col and collagen substrates, Laemmli Sample Buffer (Bio-Rad) was used to lyse the proteins, and the BCATM Protein Assay Kit (Thermo Scientific) was used to determine the concentration of the extracted proteins. Then, 60 mg of proteins was electrophoresed by SDS-PAGE and transferred to a PVDF membrane (Millipore, Corporation, MA) for detection of the target proteins: NC (1:800, Abcam) and Cx-43 (1:8000, Abcam). The housekeeping GAPDH (1:2000, Santa Cruz Biotechnology) was detected as a normalized control. After washing, the membranes were incubated with the appropriate HRP-conjugated secondary antibodies (1:2000, Invitrogen) and labeled proteins were visualized using the ECL chemiluminescence reagent. The band intensities were analyzed with ImageJ software.

### Intracellular Calcium Transient Measurements

NRVMs were incubated with 10 mM fluo-4 AM (Invitrogen) and 0.1 % Pluronic F-127 (Sigma) for 30 min at 37 °C while protected from light and washed in HBSS (Gibco) on days 5 and 7. The calcium transients were imaged under a Nikon Eclipse Ti-E confocal imaging system and analyzed with Volocity software. Fluorescent signals (*F*) were normalized to the basal cell fluorescence after dye loading (*F*_0_). The calibrated pseudo-ratio equation was as follows: [Ca^2+^]_i_ = *K*_d_(*F*/*F*_0_)/(*K*_d_/[Ca]_i − rest_ + 1 − *F*/*F*_0_) with *K*_d_ = 1100 nmol/l and [Ca^2+^]_i − rest_ = 100 nmol/l.

### BrdU Staining of Cardiac Fibroblasts

Bromodeoxyuridine (BrdU) staining was used to assess the proliferation of cardiac fibroblasts cultured on CNH-Col and collagen substrates. On days 1, 3, and 7, BrdU reagents (20 μmol/ml) were incorporated into the medium of cardiac fibroblasts and incubated for 4 h. After fixing, the cardiac fibroblasts were denaturized with hydrochloric acid (2 mol/l) and then neutralized with sodium borate (0.1 mol/l) for 12 min at room temperature. Mouse monoclonal anti-BrdU (1:100, Abcam) was used to stain proliferative cells at 4 °C overnight. Alexa Fluor 548-conjugated secondary antibodies (1:500, Invitrogen) were utilized to conjugate with the primary antibody. Then, the cells were counterstained with DAPI in D-PBS. Finally, the cells were visualized under a fluorescence microscope in 10 randomly selected fields for each group. The images were analyzed by ImageJ software and Image-Pro Plus 6.0.

### Statistical Analysis

Statistical analysis was performed by one-way ANOVA analysis followed by Tukey’s post hoc test using GraphPad Prism Software (V.6). The data were reported as the mean ± standard deviation (SD). *P* values <0.05 were considered statistically significant (*P < 0.05, **P < 0.01, ****P < 0.001, ***P < 0.0001).

## Results

### CNH-Col Material Characterization

#### Morphological Characteristics of CNH-Col Material

**Fig. 1:**
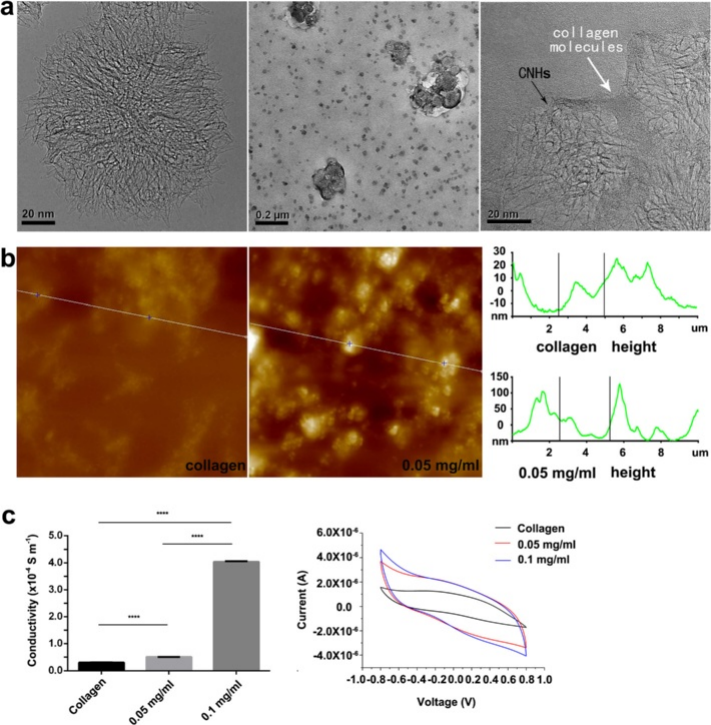
Morphological characteristics and conductivity of CNH-Col. From left to right: **a** TEM images of pristine CNH aggregates, 0.05 mg/ml CNH-Col conjugate, and high-magnification TEM image of CNH aggregates coated by collagen molecules; **b** AFM images showing the surface roughness of collagen and 0.05 mg/ml CNH-Col substrates (10 μm × 10 μm); and **c** conductivity and cyclic voltammetry curves of collagen and CNH-Col. Data are means ± SDs, *n* = 3

Pristine CNHs form spherical aggregates, as shown in Fig. 1a. After sonication under our experimental conditions, CNH aggregates were homogeneously dispersed in the collagen matrix. Small agglomerates containing only a few CNH aggregates were observed by TEM, and the CNH aggregates are coated with collagen molecules (Fig. 1a), which reveals the good affinity between the CNHs and collagen. AFM showed that the surface roughness of the CNH-Col substrate was higher compared with the collagen control (Fig. 1b).

#### Electroactivity and Conductivity of CNH-Col

There were significant differences in conductivity among the collagen and the 0.05 and 0.1 mg/ml CNH-Col groups (Fig. 1c and Additional file 1: Table S1). Notably, the conductivity of the 0.1 mg/ml CNH-Col hybrid reached 10^−4^ S/m. Cyclic voltammetry curves are shown in Fig. 1c. The ideal pseudo-capacitive nature is shown by the symmetry of the curves. The closed curve area is positively correlated with the capacitance. The capacitance of collagen is quite low. The closed curve area of the CNH-Col hybrids was larger than that of collagen. These results indicate that when CNHs are incorporated, the conductivity and capacitance of the growth substrates are improved.

### Viability, Proliferation, and Organization of NRVMs on Different Substrates

**Fig. 2:**
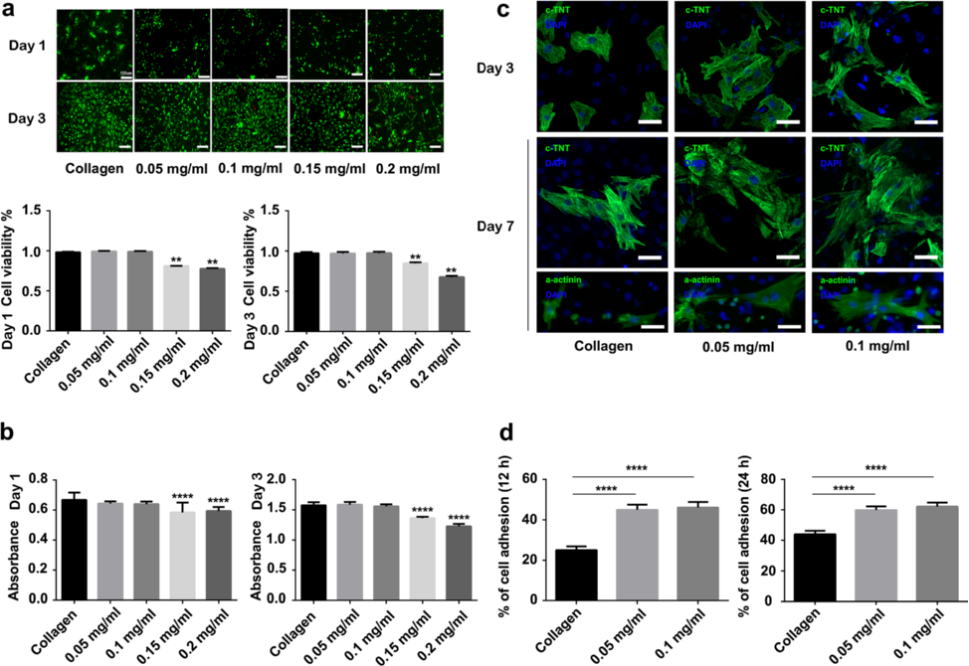
NRVM viability, organization, and adhesion rate on collagen and CNH-Col substrates. **a** NRVMs were stained with calcein-AM (*green*)/ethidium homodimer (*red*) on days 1 and 3 (*bars* = 100 um). The histogram shows that compared with the collagen group, the 0.15 and 0.2 mg/ml CNH-Col groups exhibited significant cytotoxicity to NRVMs on both days 1 and 3. **b** The CCK-8 assay shows significant differences between the collagen and CNH-Col substrates with 0.15 and 0.2 mg/ml CNHs. However, there was no significant difference between the collagen and CNH-Col substrates with 0.05 and 0.1 mg/ml CNHs. **c** Immunostaining of troponin T and sarcomeric α-actinin of NRVMs on days 3 and 7 (*bars* = 40 um). **d** There was a significant difference in the NRVM adhesion rate between the collagen and CNH-Col substrates with 0.05 and 0.1 mg/ml CNHs at both 12 and 24 h. Data are means ± SDs, *n* = 3

To evaluate the biocompatibility of CNH-Col substrates and to determine the optimal concentration for NRVM cultivation, LIVE/DEAD staining and the CCK-8 assay were carried out on days 1 and 3. The LIVE/DEAD staining (Fig. 2a) showed that there was no significant difference in cytotoxicity of NRVMs between the CNH-Col substrate groups (0.05 and 0.1 mg/ml) and collagen groups. But higher concentrations (0.15 and 0.2 mg/ml) of the CNH groups induced significant cytotoxicity to NRVMs seeded on substrates compared with the collagen group at both time points (**P < 0.01). The CCK-8 assay (Fig. 2b) showed the same trend as the LIVE/DEAD assay in that there was no significant difference among the collagen and the 0.05 and 0.1 mg/ml groups, but the 0.15 and 0.2 mg/ml groups exerted significant cytotoxicity and inhibited the proliferation of NRVMs compared with the collagen group (****P < 0.0001) on both days 1 and 3. Therefore, 0.05 and 0.1 mg/ml CNH-Col were chosen as growth substrates for further research.

Immunofluorescence staining with c-TnT and α-actinin showed that the NRVMs cultured on substrates were detected with green light (Fig. 2c). The CNH-Col groups tended to form syncytium with a more homogeneous alignment, more extensive sarcomeric structures along the cell longitude, and better cross-striation compared with the collagen group. Immunofluorescence staining of α-actinin in NRVMs for identification on day 7 is shown in Additional file 1: Figure S1, and more than 90 % of cells were identified as NRVMs.

### Cell Adhesion

To estimate whether CNH-Col substrates are suitable for NRVM growth, the effect of the substrates on the adhesion of NRVMs was estimated. There were significant differences between the CNH-Col groups and the collagen group (****P < 0.0001) at both 12 and 24 h, but no significant difference between the 0.05 and 0.1 mg/ml groups (Fig. 2d). The data indicate that the incorporation of CNHs enhances the adhesion of NRVMs to the substrates.

### Effect of CNHs on Expression, Distribution, and Function of Cx-43 and NC

**Fig. 3:**
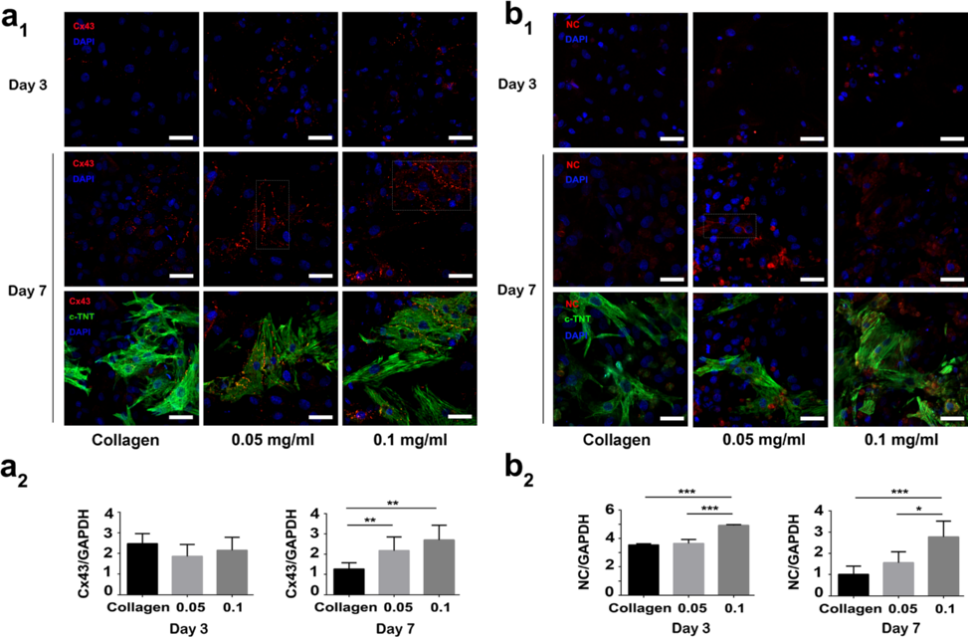
Expression and distribution of Cx-43 and NC proteins in NRVMs. **a**_**1**_,** b**_**1**_ Immunostaining of Cx-43 and NC in NRVMs (*bars* = 40 um). **a**_**2**_,** b**_**2**_ Western blotting revealed that Cx-43 and NC expression differed significantly between collagen and CNH-Col substrates at different stages. Data are means ± SDs, *n* = 3

NRVMs are one kind of typical cells with strong electric conduction and contraction. To assess the effect of CNHs on NRVM differentiation and maturation, Cx-43 and NC, which were related to electrophysiological properties and the mechanical properties, were measured by immunofluorescent staining and western blotting on days 3 and 7 (Fig. 3 and Additional file 1: Figure S2). On day 3, Cx-43 showed a trend of increased accumulation in CNH-Col groups (0.05 and 0.1 mg/ml) compared with that in the collagen group, but only in the 0.1 mg/ml group was the NC accumulation visibly increased. At this stage, Cx-43 was dispersed in the cytoplasm and cytomembrane as a mottled pattern. NC was slightly scattered in the cytoplasm, and perinuclear region western blotting also showed comparable results, such that the NC quantification normalized by GAPDH in the 0.1 mg/ml CNH-Col group was significantly higher than that in the 0.05 mg/ml CNH-Col and collagen groups (***P < 0.001). However, the Cx-43 expression quantities in the three groups showed no significant difference. At day 7, the Cx-43 and NC were developed and showed accumulations in the intercellular region of cells in the CNH-Col groups. Cx-43 appeared to specifically gather in a linear distribution between cells in the CNH-Col groups, where they could perform their electrical conduction function. However, in the collagen group, most of the Cx-43 was distributed in the cytoplasm. NC was primarily observed in the cytoplasm as a mottled or linear pattern in all the three groups. However, the NC distribution area in the 0.1 mg/ml CNH-Col group was wider than that in the 0.05 mg/ml and collagen groups. Western blotting also showed that the Cx-43 quantification normalized by GAPDH in the collagen group was significantly lower than that in the 0.1 and 0.05 mg/ml CNH-Col groups (**P < 0.01). At the same stage, the NC quantification in the 0.1 and 0.05 mg/ml CNH-Col groups was significantly higher than that in the collagen group (***P < 0.001, *P < 0.05).

**Fig. 4:**
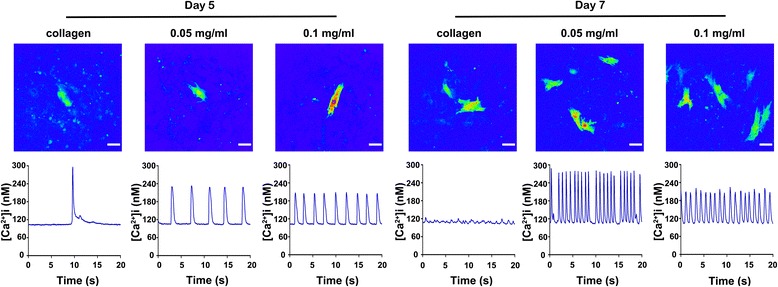
Spontaneous calcium transients in NRVMs cultured on CNH-Col substrates and collagen on days 5 and 7. (*Top*) Representative confocal laser microscopy rainbow images. (*Bottom*) Plots representing intracellular calcium transient changes with time. *Bars* = 40 μm, *n* = 3

To further estimate the effect of CNHs on protein function, spontaneous calcium transients on NRVMs were recorded using fluo-4 AM. As shown in Fig. 4, the NRVMs cultured on the CNH-Col substrates exhibited more uniform, stable rhythmic Ca^2+^ fluctuations and higher Ca^2+^ amplitudes compared with those cultured on the Col substrates on both days 5 and 7.

These data indicate that CNHs can promote the expression and intercellular distribution of electrical and mechanical proteins and functional maturation of NRVMs.

### Effect of CNHs on Cardiac Fibroblasts

**Fig. 5:**
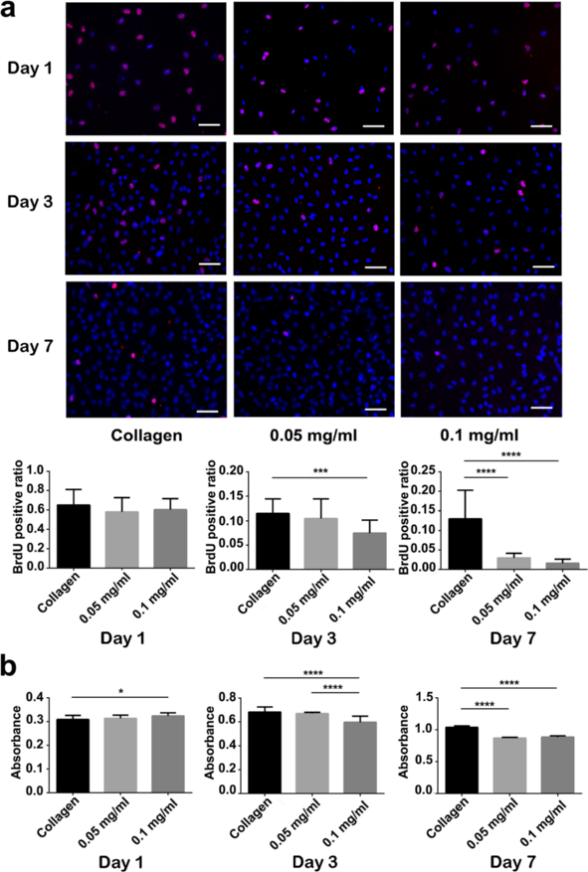
Proliferation of cardiac fibroblasts cultured on collagen and CNH-Col substrates on days 1, 3, and 7. **a** BrdU staining, where the *red* immunofluorescence represents proliferative cardiac fibroblasts (*bars* = 60 um). The histogram shows that there are significant differences in the BrdU-positive rate between the collagen and CNH-Col substrates at different stages, especially for the 0.1 mg/ml CNH-Col group. **b** The CCK-8 assay shows that there are significant differences in the absorbance of cardiac fibroblasts cultured on collagen and CNH-Col substrates at different stages. Data are means ± SDs, *n* = 3

As is known, fibrosis caused by cardiac fibroblasts is the major cause of cardiac remodeling after myocardial infarction [12] and progressively impaired ventricular function [20]. BrdU immunofluorescent (Fig. 5a) staining and the CCK-8 (Fig. 5b) assay were carried out to determine the effect of CNHs on the proliferation of cardiac fibroblasts. On day 1, there was no significant difference between the collagen group and the CHN-Col groups with respect to the BrdU-positive ratio. At this stage, more than 50 % of the cardiac fibroblasts were under proliferation. However, the CCK-8 assay showed that the absorbance in the 0.1 mg/ml CNH-Col group was significantly higher than that in the collagen group (*P < 0.05). On day 3, the BrdU-positive cells accounted for less than 10 % in the 0.1 mg/ml CNHs-Col group, significantly lower than in the collagen group (***P < 0.001). The CCK-8 assay showed a significantly decreased absorbance in the 0.1 mg/ml group compared with the other two groups (****P < 0.0001). On day 7, in the two CNH-Col groups, the BrdU-positive cells accounted for less than 5 %, which was significantly lower than that in the collagen group (****P < 0.0001). The CCK-8 assay result was in accordance with that of the BrdU staining. These data demonstrate that under the present cultivation conditions, cardiac fibroblasts can be inhibited by CNHs at concentrations of 0.1 and 0.05 mg/ml.

## Discussion

In this study, we have developed and characterized a new scaffold based on natural collagen with CNHs for CTE. Our results demonstrated that this new mixed scaffold was able to enhance the differentiation and maturation of NRVMs, and promote the expressions of electrical and mechanical proteins (Cx-43 and NC). Moreover, it could simultaneously inhibit the viability and proliferation of cardiac fibroblasts.

The collagen we utilized in our study is an important component in the heart, and it exhibits minimal inflammation and foreign body reaction with excellent biocompatibility and biodegradability, suitable for growing engineered tissues. But some limitations of natural scaffolds for CTE still exist such as instable mechanical properties or weak electrical conductivity. To solve these problems, several independent groups have previously demonstrated that carbon nanomaterials exert inspiring biological effects on seeded cells for CTE [8, 10, 14, 21–24]. In our study, we first integrate CNH with collagen to optimize the properties of scaffolds for CTE. Owing to not having a metal catalyst and their unique structure, CNHs exhibit distinct properties compared with other carbon nanomaterials for CTE. Our results demonstrated that this new scaffold did enhance the differentiation and maturation of NRVMs.

As gap junctions and adherens junctions are the foundation of intercellular communication and synchronous contraction, we further examined Cx-43 and NC expression and localization as markers for the differentiation and maturation. We found that the 0.1 mg/ml CNH-Col group exhibited significantly increased Cx-43 and NC expression compared with the collagen group on both days 3 and 7. Multiple mechanisms may be responsible for this behavior. On the one hand, our previous work showed that CNH aggregates possess satisfactory conductivity at approximately 10^5^ S/m [23]. When incorporated into collagen, the conductivity of the CNH-Col hybrids was significantly increased compared with collagen. The increased conductivity of CNH-Col can provide a continuous transport path for electrons, which is beneficial to excited cells (cardiac myocytes and neurons) [24]. A previous study reported that the conductivity of native myocardium ranges from 1.6 × 10^−1^ to 5 × 10^−3^ S/m [25], which is higher than that of the CNH-Col hybrids, but there is a lack of literature on the conductivity of human epicardium tissue. However, the substrates are intended to be applied as cardiac patches on the epicardium, which has the conductivity lower than that of the myocardium. The increased capacitance of the CNH-Col hybrids can provide a pseudo-container for electron enrichment, which is beneficial for reaching the threshold value of NRVM excitement. On the other hand, carbon nanomaterials present a unique rough nanostructure [8, 26–29]. We found that the incorporation of CNHs in collagen increased the roughness and contact area of the substrates. From the above, we speculate that the improvement of the conductivity and surface roughness of the substrates favors the formation of intercellular tight interactions and enhances the connection between the NRVMs and the substrates by providing a desired artificial extracellular matrix and by regulating protein expression and functioning.

Carbon nanomaterials have been reported to display cell-specific characteristics that benefit cardiomyocytes and neurons, whereas fibroblasts and glial cells remain unchanged [14, 20, 28]. It has been reported that unmodified SWNHs exhibit different interaction mechanisms in human normal liver cell lines and hepatoma cell lines [30]. We verified that the 0.1 mg/ml CNH-Col group could significantly inhibit cardiac fibroblast proliferation on days 1, 3, and 7 while also maintaining the NRVM viability.

## Conclusions

In this study, we found that the conductivity and surface roughness of the CNH-Col substrates were significantly increased compared with those of pure collagen. Both 0.05 and 0.1 mg/ml CNH-Col can up-regulate Cx-43 and NC expression and increase spontaneous calcium transients compared with collagen. At the same time, CNHs can inhibit cardiac fibroblast proliferation. Thus, CNH may be a promising scaffold material to meet the requirements of CTE.

## Additional file

Additional file 1: Figure S1–S2 and Table S1.
**Figure S1.** Immunofluorescence staining of α-actinin in NRVMs for identification on day 7 (bar = 30 um). **Figure S2.** Cx-43 and NC expression. Representative western blotting bands of Cx-43 and NC proteins in NRVMs cultured on CNH-Col and collagen substrates on days 3 and 7, *n* = 3. **Table S1.** Summary of the electrical conductivity of CNH-Col substrates (S/m). (DOCX 343 kb)

## Abbreviations

AFM: atomic force microscope
BrdU: bromodeoxyuridine
CCK-8: Cell Counting Kit-8
CNH-Col: carbon nanohorn-collagen
CNHs: carbon nanohorns
CTE: cardiac tissue engineering
Cx-43: connexin-43
DMEM: Dulbecco’s modified Eagle’s medium
FBS: fetal bovine serum
NC: N-cadherin
NRVMs: neonatal rat ventricular myocytes
TEM: transmission electron microscopy
