# Supplementary material for: Carbon Nanohorns Promote Maturation of Neonatal Rat Ventricular Myocytes and Inhibit Proliferation of Cardiac Fibroblasts: a Promising Scaffold for Cardiac Tissue Engineering
Source: Nanoscale Res Lett. 2016 Jun 4;11:284. doi: 10.1186/s11671-016-1464-z (PMC4893350; doi:10.1186/s11671-016-1464-z)
Supplement: Additional file 1: Figure S1–S2 and Table S1. — Figure S1. Immunofluorescence staining of α-actinin in NRVMs for identification on day 7 (bar = 30 um). Figure S2. Cx-43 and NC expression. Representative western blotting bands of Cx-43 and NC proteins in NRVMs cultured on CNH-Col and collagen substrates on days 3 and 7, n = 3. Table S1. Summary of the electrical conductivity of CNH-Col substrates (S/m). (DOCX 343 kb) [file 11671_2016_1464_MOESM1_ESM.docx]

**Additional file 1**


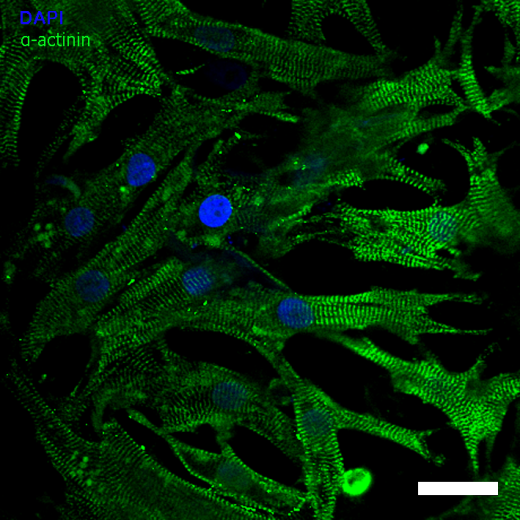


Fig. S1. Immunofluorescence staining of α-actinin in NRVMs for identification on day 7 (bar = 30 um).


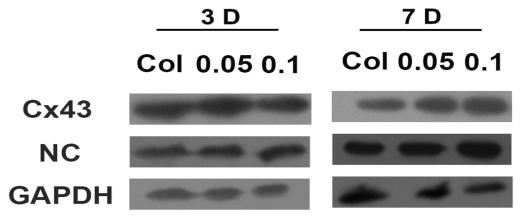


Fig. S2. Cx-43 and NC expression. Representative western blotting bands of Cx-43 and NC proteins in NRVMs cultured on CNH-Col and collagen substrates on days 3 and 7. n=3.

**Table S1: Summary of the electrical conductivity of CNH-Col substrates.** （S.m^-1^）

| CNHs (mg/ml) Mean Standard deviation |
| --- |
| 0 2.977x10^-5^ 3.301x10^-7^  0.05 5.037x10^-5^ 4.127x10^-7^  0.1 4.039x10^-4^ 1.982x10^-6^ |
